# Supplementary material for: A Humanized Yeast Phenomic Model of Deoxycytidine Kinase to Predict Genetic Buffering of Nucleoside Analog Cytotoxicity
Source: Genes (Basel). 2019 Sep 30;10(10):770. doi: 10.3390/genes10100770 (PMC6826991; doi:10.3390/genes10100770)
Supplement: Supplementary file 1 [file genes-10-00770-s001.zip › Additional_Files/Additional_File7_GOTermHeatmaps.zip!/Additional_File7_GOTermHeatmaps/ubiquitin-dependent_protein_catabolic_process_via_the_multivesicular_body_sorting_pathway.pdf]

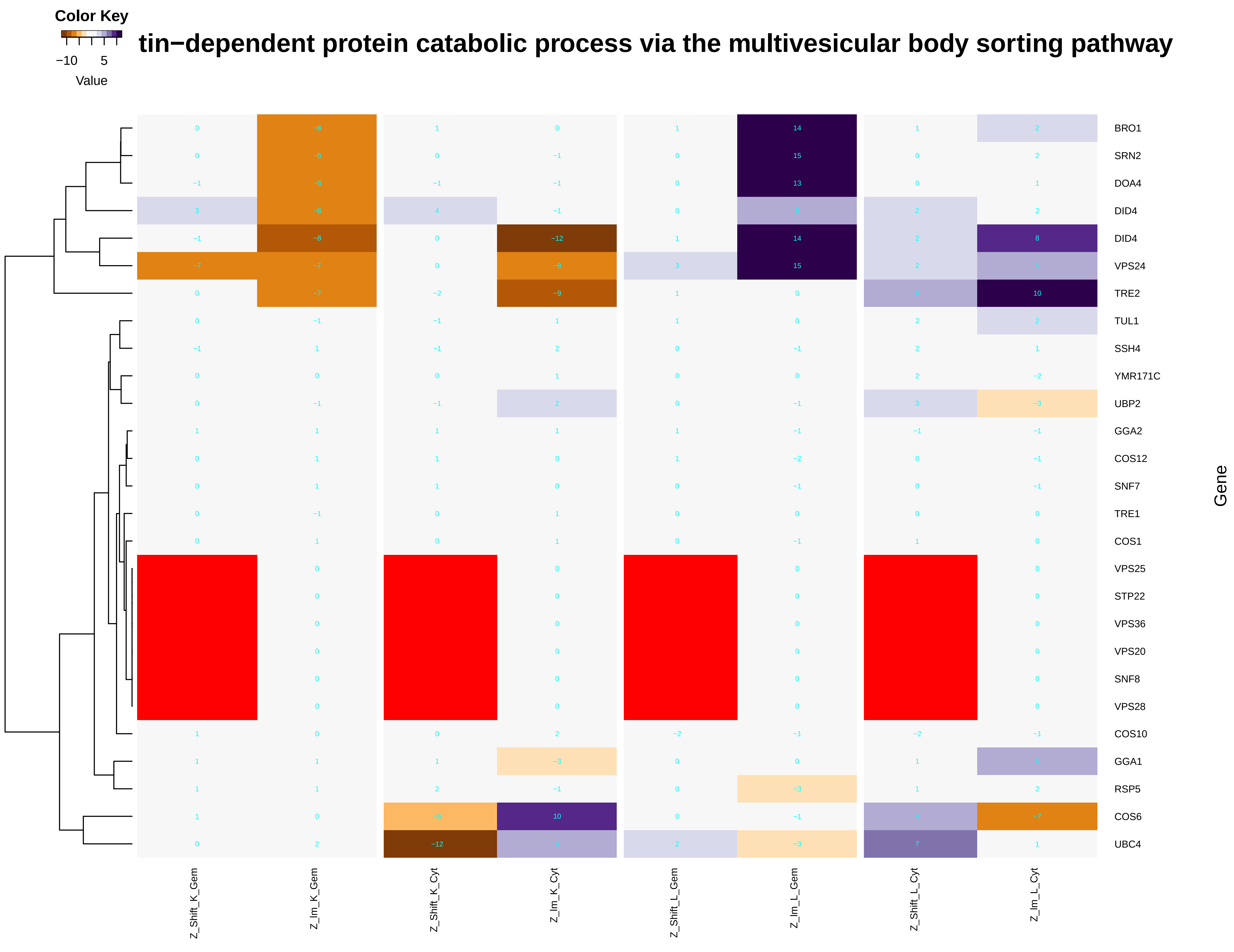

Color Key
−10
5
Value
tin−dependent protein catabolic process via the multivesicular body sorting pathway
BRO1
SRN2
DOA4
DID4
DID4
VPS24
TRE2
TUL1
SSH4
YMR171C
UBP2
GGA2
COS12
SNF7
TRE1
COS1
VPS25
STP22
VPS36
VPS20
SNF8
VPS28
COS10
GGA1
RSP5
COS6
UBC4
Gene
Z_Shift_K_Gem
Z_Im_K_Gem
Z_Shift_K_Cyt
Z_Im_K_Cyt
Z_Shift_L_Gem
Z_Im_L_Gem
Z_Shift_L_Cyt
Z_Im_L_Cyt
